# Nanopore long-read next-generation sequencing for detection of mitochondrial DNA large-scale deletions

**DOI:** 10.3389/fgene.2023.1089956

**Published:** 2023-06-29

**Authors:** Chiara Frascarelli, Nadia Zanetti, Alessia Nasca, Rossella Izzo, Costanza Lamperti, Eleonora Lamantea, Andrea Legati, Daniele Ghezzi

**Affiliations:** ^1^ Unit of Medical Genetics and Neurogenetics, Fondazione IRCCS Istituto Neurologico Carlo Besta, Milan, Italy; ^2^Department of Pathophysiology and Transplantation (DEPT), University of Milan, Milan, Italy

**Keywords:** mtDNA, long reads, oxford nanopore, MinION, macrodeletion, multiple deletions, complex-rearrangements, structural variants

## Abstract

Primary mitochondrial diseases are progressive genetic disorders affecting multiple organs and characterized by mitochondrial dysfunction. These disorders can be caused by mutations in nuclear genes coding proteins with mitochondrial localization or by genetic defects in the mitochondrial genome (mtDNA). The latter include point pathogenic variants and large-scale deletions/rearrangements. MtDNA molecules with the wild type or a variant sequence can exist together in a single cell, a condition known as mtDNA heteroplasmy. MtDNA single point mutations are typically detected by means of Next-Generation Sequencing (NGS) based on short reads which, however, are limited for the identification of structural mtDNA alterations. Recently, new NGS technologies based on long reads have been released, allowing to obtain sequences of several kilobases in length; this approach is suitable for detection of structural alterations affecting the mitochondrial genome. In the present work we illustrate the optimization of two sequencing protocols based on long-read Oxford Nanopore Technology to detect mtDNA structural alterations. This approach presents strong advantages in the analysis of mtDNA compared to both short-read NGS and traditional techniques, potentially becoming the method of choice for genetic studies on mtDNA.

## Introduction

Human mitochondria are double-membrane, intracellular organelles, whose primary function is to produce energy, in the form of ATP, by aerobic respiration in the oxidative phosphorylation system (OXPHOS). Mitochondria are crucial for normal cellular function as they are responsible for many other important cellular processes (Giacomello et al., 2020; Osellame et al., 2012). These organelles contain their own genome with a modified genetic code. The mammalian mitochondrial genome is transmitted exclusively through the female germ line. The human mitochondrial DNA (mtDNA) is a double-stranded, circular molecule of 16,569 bp and contains 37 genes coding for 2 rRNAs, 22 tRNAs and 13 subunits of the OXPHOS enzyme complexes (Taanman, 1999). It is present in multiple copies, ranging from tens to thousands, in each cell. Homoplasmy is the condition in which all the mtDNA copies are identical. Conversely, mtDNA molecules with two different sequences (usually the wild type and a mutant one) can exist together in a single cell, a condition known as heteroplasmy. When mtDNA pathogenic variant levels reach heteroplasmic percentages higher than a certain threshold, mitochondrial dysfunctions can occur leading to a number of clinical manifestations (Taylor and Turnbull, 2005). For point mutations, threshold is typically 60%–95% depending on the variant and cell type, even though heteroplasmic variants <50% can cause pathogenic effects (McCormick et al., 2020). In addition, heteroplasmy percentages are different in various tissues and may change over time, further complicating the definition of a strict correlation between heteroplasmy and severity of the disease. For large-scale mtDNA deletions, the degree of heteroplasmy in muscle may be highly variable with some affected subjects presenting low percentages (∼20% in schröder et al., 2000; ∼40% in Grady et al., 2014).

Mitochondrial diseases (MD) form a group of rare, genetically and clinically heterogeneous conditions characterized by impairment of the mitochondrial respiratory chain. The pathophysiology of MD is complex and involves pathogenic variants in genes, belonging to either nuclear DNA (nDNA) or mtDNA, that encode RNAs/proteins involved in mitochondrial function (Moggio et al., 2014). Pathogenetic mtDNA variants can affect the structural subunits of the respiratory chain or the mitochondrial protein synthesis machinery. Hundreds of different point mutations and large-scale mtDNA rearrangements have been shown to cause disease (Mitomap, 2023, https://www.mitomap.org). In particular, variants in mtDNA causing MD can be classified into three types: point mutations in genes encoding structural proteins (variants in the 13 mtDNA genes encoding OXPHOS subunits), point mutations in genes involved in protein synthesis (i.e., variants in mitochondrial tRNA or rRNA), or mtDNA rearrangements (for example, sporadic, single large-scale mtDNA deletions) (Gorman et al., 2016).

Devising a diagnostic algorithm that encompasses all MD is really complicated especially because of the diverse clinical manifestations of disease and heterogeneous genetic bases. Next-generation sequencing (NGS) has revolutionized the diagnosis and discovery of new disease genes responsible for heterogeneous disorders, including mitochondrial diseases (Legati et al., 2016; Stenton and Prokisch, 2020). Approaches based on short-read NGS are currently used for detection of single point variants and evaluation of their heteroplasmy level, while their use for identification of mtDNA rearrangements is not well standardized. Using short-read NGS, DNA is fragmented into low molecular weight species, typically <0.5 kb. As a result, the genomic contiguity found in circular mtDNA molecules get lost, making more difficult to determine structural changes or complex rearrangements (Greer et al., 2017). Furthermore, most short-read NGS approaches for mtDNA analysis rely on the sequencing of mtDNA PCR amplicons, a strategy that can introduce bias in the detection of large deletions or complex rearrangements (Goudenège et al., 2019; Basu et al., 2020; Legati et al., 2021). Few methods for quantifying deletions in mtDNA by short-read NGS have been published [e.g., LostArc (Lujan et al., 2020)].

MtDNA single large deletions are the genetic defect present in a subset of MD including Pearson syndrome (an infantile multisystem disorder characterized by sideroblastic anemia and pancreatic insufficiency), Kearns-Sayre syndrome (an early-onset neuromuscular disorder with retinopathy, ataxia, hearing loss, ptosis, muscle weakness) and chronic Progressive External Ophthalmoplegia or PEO (mitochondrial myopathy characterized by adult-onset ptosis and impaired eye movements). In affected children, mtDNA deletions are often detectable in all specimens (including blood and urine), whereas the analysis of muscle biopsy is needed in adult patients (Goldstein & Falk, 2003).

In addition to primary single large-scale mtDNA deletions, mitochondrial myopathies (MM) can be caused by pathogenic variants in nuclear genes encoding the mtDNA maintenance machinery, that can lead to secondary multiple mtDNA deletions. Also in this case, mtDNA defective molecules can be typically identified only in muscle (El-Hattab et al., 2017).

New NGS technologies based on long-reads have been released (PacBio; Oxford Nanopore Technology-ONT) (Macken et al., 2021). These sequencing tools, called “third generation” NGS, can produce reads of several kb in length (short reads are usually ∼150–300 bp in length, allowing at most up to 800 bp-long reads), enabling the entire mitochondrial genome to be sequenced in one read. Despite long-read sequencing of mtDNA is in its infancy, it has the potential to become an “all-in-one” solution for mtDNA genetic testing. Indeed, it could enable the identification of point variants and large-scale rearrangements together with quantification of their heteroplasmy, through PCR-free approaches (Wong, 2013). The sequencing of entire mitochondrial genomes with ONT technology, with or without mtDNA enrichment steps, has been made possible by the development of a few protocols (Zascavage et al., 2019). Recent ONT approaches promise improved resolution of homopolymeric regions of mtDNA and are less work intensive than other NGS protocols. An initial attempt to use ONT sequencing in the clinical setting (for identification of mtDNA deletions) yielded promising results (Wood et al., 2019).

The MinION is a portable device allowing rapid, real-time long read sequencing of nucleic acids based on nanopore technology. It works identifying DNA bases by measuring the changes in electrical conductivity generated as DNA strands pass through a biological pore; these current changes are recorded producing reads in real-time, each read corresponding to a single strand of DNA. Eventually, the signals stored are processed by base-calling algorithms to decode the nucleotide sequences into FASTQ files, the file format used for most downstream bioinformatics analyses.

In this study, we tested two protocols based on long-read ONT sequencing to detect mtDNA structural alterations by using the MinION device. We exploited muscle samples from patients with known genetic defects of mtDNA, either single mtDNA macro-deletions or multiple mtDNA deletions, detected by traditional techniques. We compared the ONT findings for the analysis of mtDNA to both conventional approaches and short-read NGS.

## Methods

### Patients’ cohort selection

During the past 15 years the Unit of Medical Genetics and Neurogenetics (Fondazione IRCCS Istituto Neurologico Besta) collected a large cohort of mitochondrial myopathies (MM) patients on the basis of histological alterations in the muscle biopsy and a clinical presentation suggestive for MM, with ophthalmoplegia and/or ptosis. For this study, we selected mitochondrial disease patients, previously diagnosed by Southern blot (SB): 9 carrying mtDNA single large deletions (del1, del2, del3, del4, del5, del6, del7, del8, and del9) and 4 carrying mtDNA multiple deletions (multidel1, multidel2, multidel3, and multidel4) (Table 1). Moreover, we selected 3 control patients (ctrl1, ctrl2, and ctrl3) with a clinical presentation not indicating mitochondrial involvement, and with muscle biopsy previously analyzed by SB, with no mtDNA structural alterations.

**TABLE 1:** Details about the molecular mtDNA defects in the patients analyzed in the present study.

*Sample code*	*Deletion*				*KIT* (*library preparation method*)* - Enzyme (endonuclease for mtDNA linearization*)	*Heteroplasmy (Nanopore sequencing)* (%)
	*Start*	*End*	*Del size (bp)*	*Heteroplasmy assessed by SB or qPCR^a^*		
*Del1*	7,634	13,956	6,322	50%	LIG—BamHI	52
					RAPID—transposase	38
*Del2*	8,482	13,446	4,964	35%	LIG—BamHI^b^	16
					LIG-PvuII	36
					RAPID—transposase	18
*Del3*	7,343	15,602	8,259	40%	LIG—BamHI^b^	7
					LIG- PvuII	57
*Del4*	10,955	15,544	4,589	50%	LIG-BamHI^b^	26
*Del5*	8,482	13,460	4,978	50%	LIG—BamHI^b^	59
					LIG- PvuII	69
*Del6*	11,570	15,573	6,997	60%	LIG—BamHI^b^	10
*Del7*	7,829	14,826	6,997	50%	LIG-BamHI^b^	22
					LIG-PvuII	71
*Del8*	12,714	15,862	3,148	50%	LIG—BamHI^b^	2
					LIG- PvuII	43
*Del9*	6,341	14,005	7,664	30%	LIG—BamHI^b^	29
					LIG-PvuII	40

*Sample code*	*Disease Gene*	*Genomic Variant*	*AA change*	*KIT*—*Enzyme*	
*Multidel1*	*TWNK*	chr10:102749067 T>C (heterozygous)	p.Ile367Thr	RAPID—transposase	
*Multidel2*	*TWNK*	chr10:102749592 G>A (heterozygous)	p.Glu479Lys	RAPID—transposase	
*Multidel3*	*C1QBP*	chr17:5336700 G>C (homozygous)	p.Phe204Leu	RAPID—transposase	
*Multidel4*	Not known	Not known	Not known	RAPID—transposase	

^a^ SB, southern blot; qPCR, quantitative PCR. LIG: ligation sequencing kit, RAPID: rapid sequencing kit.

^b^ Restriction site within the deletion (underestimated heteroplasmy). Genomic variant: information regarding variant position in the nuclear genome (hg19) and nucleotide change. AA, change: effect of the genomic variant in the amino acid sequence of the protein encoded by the disease gene.

For samples del4 and del6 there was not enough material for a second analysis using PvuII restriction enzyme. Further information regarding reads parameters per sample are reported in Supplementary Table S1

### DNA extraction and quantification

The extraction of the DNA from muscle biopsies was performed using the Phenol-chloroform DNA purification method, followed by DNA quantification by Qubit Fluorometer (Thermo Fisher Scientific Inc.) and quality parameters check by Nanodrop (Thermo Fisher Scientific). DNA concentration was estimated by Qubit quantification with the following protocol.

1. Two assay tubes for the standards were set and one assay tube for each sample.
2. Qubit working solution was prepared by diluting the Qubit reagent 1:200 in Qubit buffer. Then, 200 μL of working solution were prepared for each standard and sample.
3. Assay tubes were prepared according to the values reported in the table present in the Qubit protocol [Thermo Fisher Scientific, 2023 protocol link in WEB resources].
4. All tubes were vortexed for 2-3 s.
5. Tubes were incubated for 2 min at room temperature.
6. Eventually, tubes were inserted in the Qubit Fluorometer and readings were taken.

DNA quality parameters (A260/280 and A260/230 ratios) were checked by Nanodrop analyzing 1.5 µL of DNA.

### ONT library preparation

Oxford Nanopore company provides a wide range of library preparation kits which are available to suit all whole genome sequencing requirements. In particular, amplification-free kits allow direct, long-read sequencing of native DNA, eliminating the potential for PCR bias.

For this study, two different amplification-free kits for Nanopore MinION sequencing were tested, the Ligation Sequencing Kit (SQK-LSK109) and the Rapid Sequencing Kit (SQK-RAD004) (see links to protocol in WEB resources).

#### ONT ligation sequencing kit (SQK-LSK109)

This kit is recommended for users who want to optimize their sequencing experiment for throughput, in terms of read length and quantity. For highest data yields, it is recommended starting with 100–200 fmol of pure input DNA. Starting with lower amounts of input material, or impure samples, can affect library preparation efficiency and can reduce sequencing throughput. It is possible to either start with 1 μg of gDNA, quantified using the Qubit fluorometer, or 100–200 fmol of shorter-fragment input such as amplicons or cDNA. It is recommended that the DNA samples should meet the following criteria: A260/280 = 1.8 and A260/230 = 2.0–2.2. Starting with lower amounts of input material, or impure samples, can affect library preparation efficiency and can reduce sequencing. If the experiment requires long reads, it is recommended to start with full-length DNA, and fragmentation/shearing is neither advised nor required; to preserve long fragments present in the starting material. However, because of the absence of an enzymatic digestion step and the circularity of the mitochondrial genome, a linearization step needs to be introduced to properly sequence mtDNA.

##### DNA processing with BamHI or PvuII restriction enzymes

Linearization of the circular mitochondrial genome was performed by the cutting of restriction enzymes, either BamHI (as previously described (Bicci et al., 2021) or PvuII (currently used in our laboratory for Southern blot analysis). We suggest starting with 1,300 ng of DNA, an input around 20%–30% higher than the quantity recommended in the ligation protocol (1,000 ng). Indeed, once we quantified DNA amount after the digestion, we saw a decrease in final quantity comparable to the suggested increment of starting DNA material. The protocol steps we followed to perform the mtDNA enzymatic processing are reported below.

For BamHI enzymatic digestion:

1. Add in a 0.2 µL Tube: 32.5 µL of H_2_O, 5 µL of Buffer3, 0.5 µL of BSA, 10 µL of DNA, 2 µL of BamHI enzyme (for a total of 50 µL of reaction volume).
2. Once the reaction mix is ready, put the tube in an incubator for 1 h at 37°C degrees.

For PvuII enzymatic digestion:

1. Add in a 0.5 µL Tube: 60.5 µL of H_2_O, 8 µL of Buffer10x, 10 µL of DNA, 1.5 µL of PvuII enzyme (for a total of 80 µL of reaction volume).
2. Once the reaction mix is ready, put the tube in a bath incubator for 7 h at 37°C degrees.

##### DNA purification by AMPure beads

Eventually, after digested DNA was quantified and checked for quality parameters, we suggest performing a clean-up using Agentcourt AMPure XP beads (Beckman Coulter, 2016):

1. Agencourt AMPure XP bottle was shacked to resuspend any magnetic particles that may have settled. Then Agencourt AMPure XP beads were added according to the sample reaction volume shown in the official protocol chart [Beckman Coulter, protocol link in WEB resources].
2. The volume of Agencourt AMPure XP for a given reaction was derived from the following equation: (Volume of Agencourt AMPure XP per reaction) = 1.8 × (Reaction Volume).
3. This step binds DNA fragments >100 bp to the magnetic beads. Solution reaction was mixed by pipetting. The color of the mixture appeared homogenous after mixing by pipette 10 times. Mixed samples were incubated for 5 min at room temperature for maximum recovery.
4. The reaction plate was placed onto an Agencourt SPRIPlate 96 Super Magnet plating for 2 min to separate beads from the solution. It is important to wait for the solution to clear before proceeding to the next step.
5. This step was performed while the reaction plate was placed on the Agencourt SPRIPlate 96 Super Magnet Plate; then, the cleared solution from the reaction plate was aspirated and discarded. About 5 μL of supernatant were left behind, otherwise beads would have been drawn out with the supernatant*.* Importantly, the ring of separated magnetic beads was not disturbed.
6. The next step was performed with the reaction plate placed on an Agencourt SPRIPlate 96 Super Magnet Plate. Separated magnetic beads were not disturbed and all ethanol was removed from the bottom of the well.
7. 200 μL of 70% ethanol were dispensed to each well of the reaction plate and incubated for 30 s at room temperature. Then, the ethanol was aspirated out and discarded. This procedure was repeated twice. The beads were clearly separated from alcohol, so it was not necessary to leave any supernatant behind.
8. The reaction plate was removed from the magnet plate, and then 40 μL of H_2_O added to each well of the reaction plate, mixing by pipetting 10 times. Reaction plate was then incubated for 2 min. The liquid level was high enough to contact the magnetic beads at a 40 μL elution volume. Using less than 40 μL would require extra mixing (to ensure all the liquid gets in contact with the beads).
9. The reaction plate was placed again onto an Agencourt SPRIPlate 96 Super Magnet Plate for 1 min to separate beads from the solution.
10. The eluate was transferred to a new plate or tube.

The Ligation Sequencing Kit offers a flexible method of preparing sequencing libraries from dsDNA (e.g., gDNA, cDNA or amplicons). The library preparation method is straightforward: DNA ends are repaired and dA-tailed using the NEBNext End Repair/dA-tailing module, and then sequencing adapters are ligated onto the prepared ends. Once some preliminary steps were accomplished (including the DNA processing with Restriction Enzymes and the multiplexing), as reported in the Ligation Sequencing Kit (SQK-LSK109) protocol by the Nanopore Community [https://nanoporetech.com], the library preparation started with the DNA ends repairing for the further adapter attachment step. In a 0.2 mL thin-walled PCR tube are mixed 48 µL of 1 µg input DNA, 3.5 µL NEBNext FFPE DNA Repair Buffer, 2 µL NEBNext FFPE DNA Repair Mix, 3.5 µL Ultra II End-prep reaction buffer, 3 µL Ultra II End-prep enzyme mix. Then, after purifying the reaction mix with AMPure XP beads, a volume of 61 µL of the repaired and end-prepped DNA was quantified using a Qubit fluorometer. At this point, the repaired and end-prepped DNA was ready for the sequencing adapters to be attached to DNA ends. In a 1.5 mL Tube 60 µL DNA sample from previous step, 25 µL Ligation Buffer (LNB), 10 µL NABNext Quick T4 DNA Ligase and 5 µL Adapter Mix (AMX) were mixed together. Depending on the wash buffer (LFB, Long Fragment Buffer or SFB, Short Fragment Buffer) used, the clean-up step after adapter ligation is designed to either enrich DNA fragments of more than 3 kb or purify all fragments equally. In this study we used the LFB for all the samples processed with the Ligation kit. Eventually, once the DNA library was adapter-ligated, it was ready to be loaded into the primed flow cell.

##### Multi-sample sequencing by barcode sequencing kit

According to the experimental goals, there is the possibility to use an expansion kit to amplify the number of samples to sequence in a single run. The Native Barcoding genomic DNA (EXP-NBD104-114) allows samples multiplexing to a maximum of 12 samples per run. Barcoding or multiplexing is useful when the amount of data required per sample is less than the total amount of data that can be generated from a single flow cell: this allows to pool multiple samples and sequence them together, making more efficient use of the flow-cell. The library preparation method can be considered identical to the Ligation Sequencing Kit protocol, with the additional step of barcode ligation; DNA ends are repaired and dA-tailed using the NEBNext End Repair/dA-tailing module, and then a unique dT-tailed barcode adapter is ligated on the dA-tailed template. Barcoded samples are then pooled together. Each barcode adapter also has a cohesive end which is used as a hook to bind the supplied sequencing adapters.

#### ONT rapid sequencing kit (SQK-RAD004)

This kit has a short preparation time and requires limited access to laboratory equipment. It allows to generate sequencing libraries from extracted DNA in 10 min using a simple two-step protocol. At the heart of the kit is a transposase which simultaneously cleaves template molecules and attaches tags to the cleaved ends. Rapid Sequencing Adapters are then added to the tagged ends. The kit is optimized for simplicity and speed, rather than for obtaining maximum throughput. Due to the simple nature of the workflow and the fact that little sample manipulation is required (e.g., minimal pipetting steps and no clean-ups) some very long reads can be achieved with this kit, despite the required transposase fragmentation. However, for obtaining sequencing of long reads, long fragments need to be present in the sample in the first place. The workflow is PCR-free and therefore avoids PCR bias and retains information about base modifications, which can be analyzed using specific bioinformatics tools. The Rapid Sequencing Kit recommends an input of 400 ng DNA, as for the Ligation kit. However, we suggest starting with a higher amount of input DNA (700 ng) to ensure, at the end of the standard protocol, a final DNA amount equal to or higher than the 400 ng DNA requested for starting the sequencing run. Loading less than 400 ng DNA, or the presence of highly fragmented DNA could compromise sequencing throughput and read length. For samples where only lower inputs are possible, the Low Input by PCR Sequencing Kit and Rapid Low Input by PCR Sequencing Kit are available.

Our library preparation started with the tagmentation of 700 ng input DNA diluted in 7.5 µL of H_2_O, using 2.5 µL of the Fragmentation Mix provided in the kit. The enzyme used in this step performs random cuts along the whole genome. Then 1 µL of sequencing adapters were attached to the 10 µL of DNA ends resulting from the previous step. Eventually, once the prepared DNA library was ready, flow cell priming occurred with subsequent loading of the DNA library into the MinION Flow Cell (R.9.4.1). Once the sequencing run was started by the MinKNOW software, raw data were collected from the device and converted into basecalled reads.

### Flongle flow cell

Other than loading samples on MinION Flow Cell (R9.4.1), it is possible to exploit the same library preparation protocols for both ONT Rapid and Ligation Sequencing Kits also for the Flongle Flow Cell. Flongle is an adapter for MinION or GridION that enables direct, real-time DNA sequencing on smaller, single-use flow cells. Costing nine times less than the R9.4.1 flow cell, Flongle is the quickest, most accessible solution for smaller tests and experiments. The Flongle Flow Cell requires less input material for starting a sequencing experiment and produces less output compared to the Flow Cell (R9.4.1).

A critical step during the use of the Flongle device is the loading procedure. Following the official protocol (retrievable at the Nanopore website https://community.nanoporetech.com), we observed a reduction in the number of available sequencing pores. Alternatively, we tested another loading procedure, as suggested in a special issue present in the Nanopore Community, entitled “A very gentle, relatively rapid way to load a Flongle flowcell” (community.nanoporetech.com).

In this procedure, the loading step occurs pipetting the DNA library inside the loading port in a dropwise fashion similarly to the MinION Flow Cell (R.9.4.1). This different loading approach increased the number of available sequencing pores significantly, affecting positively and significantly the sequencing output.

### Software for ONT analysis

ONT builds and provides numerous software types involved in acquisition, orchestration and analysis. We exploited the following: MinKNOW, Guppy and EPI2ME.

MinKNOW. The software runs on the host computer to which the MinION is connected. Data from MinKNOW is packaged into fast5 files, which are a customized file format based upon the .hdf5 file type. FASTQ files are also produced, containing both the basecalled sequence of DNA/RNA and its quality scores.

Guppy. This software utilizes the latest Recurrent Neural Network algorithms to interpret the signal data from ONT sequencer, and basecall the DNA or RNA passing through the pore.

EPI2ME. This software provides users with real-time analysis such as species identification, alignment workflows and other bioinformatics solutions (Amarasinghe et al., 2020).

### Sequencing on nanopore MinION

Once the MinION device is connected, MinKNOW software can be opened in order to preliminary perform hardware check (to ensure having enough space to store sequencing data collected by the software) and flow cell check, which is fundamental to check how many pores are available for the sequencing run (it is suggested to not use the R9.4.1 flow cell if less than ∼800 pores are available, or the Flongle with less than 50 pores). Once performed the aforementioned steps, the flow cell was primed using the specific reagents present in the Flow Cell Priming Kit in order to assure a better performance during the sequencing experiment. The flow cell was then loaded with DNA samples.

Finally, after the flow cell was primed and loaded, the sequencing run is manually started using MinKNOW Software. Moreover, once the run is started, it can be left ongoing for an arbitrary number of hours (ranging from 3 to 72 h), according to the number of input samples and the user desired coverage for the genomic regions of interest (for this work, our focus was on the mitochondrial genome). However, during the sequencing run, thanks to the MinKNOW Software, proceeding of the sequencing can be continuously monitored checking the pores which are effectively sequencing. MinION sequencing data were real-time collected and stored by MinKNOW Software and for each data produced, further downstream bioinformatics analyses were performed.

### Long-read downstream bioinformatic analysis

After the MinION sequencing run was stopped through the MinKNOW software, the raw data produced, FASTQ files and FAST5 files, were subjected to an accurate downstream bioinformatics analysis. For the purposes of this work a specific bioinformatics pipeline was developed to validate ONT MinION sequencing as a strategy for the detection of mtDNA structural alterations.

All the bioinformatics tools used in the following pipeline were executed from Command Line.

#### Alignment to reference genome

Read quality depends on achieving optimal translocation speed (the rate of ratcheting base by base) of the nucleic acid through the pore, which typically decreases in the late stages of sequencing runs, negatively affecting the quality (Oxford Nanopore Technologies, 2019a; Oxford Nanopore Technologies, 2019b; Oxford Nanopore Technologies, 2022; see link in WEB resources).

However, for the purposes of our work, the raw reads used for the downstream bioinformatics analyses were taken from the fastq_pass directory generated by the MinKNOW software during real time data acquisition, relying on the default quality parameters evaluated. MinKNOW has been reported to be prone to incorrectly split ultra-long reads (>50 kb) (Payne et al., 2019); however, mtDNA reads, that are shorter than 17 Kb, should be poorly affected.

Raw reads, resulting from the real time MinKNOW data acquisition, were directly aligned to the reference genome [*Homo sapiens* (human) genome assembly GRCh37 (hg19)] using Minimap2, a versatile sequence alignment tool that aligns DNA or RNA sequences against a large reference database (Li, 2018). For ∼10 kb noisy reads sequences, minimap2 is tens of times faster than mainstream long-read mappers such as BLASR, BWA-MEM, NGMLR, and GMAP. It is more accurate on simulated long reads and produces biologically meaningful alignment ready for downstream analyses. For >100 bp Illumina short reads, minimap2 is three times as fast as BWA-MEM and Bowtie2, and as accurate on simulated data (Vollger et al., 2020).

We performed minimap2 alignment taking into account spliced long-reads (unknown strand) and aligning them to the reference genome file (in our case reads were aligned to the mtDNA revised Cambridge Reference Sequence), outputting the aligned reads file in SAM format directly into a sorted BAM file, exploiting bioinformatic tools of the samtools repository. This is a tool package (written in C using htslib) for manipulating next-generation sequencing data to handle SAM, BAM and CRAM files (Danecek et al., 2021). However, deletion calling from Minimap2 aligned data was reported to have low accuracy for mtDNA deletions, particularly with increasing deletion size (Vandiver et al., 2022). Therefore, alignment with the NGMLR (Sedlazeck et al., 2013) long-read mapper was accomplished to obtain a long-read alignment best suitable for subsequent Structural Variants (SVs) call using Sniffle variant calling tool (see below) (Sedlazeck et al., 2013; Zhou et al., 2019).

In order to visualize the Minimap2 alignment, we exploited the Integrative Genomics Viewer (IGV), to appreciate coverage depth and potential genome structural alterations (Robinson et al., 2011).

Statistical bioinformatics analyses were accomplished to evaluate the MinION sequencing efficiency for the mtDNA. Statistics of the alignment were performed estimating the reads mapped exclusively on the mitochondrial chromosome respect to the ones mapped to the nuclear genome.

#### mtDNA single point mutations analysis

In order to evaluate long-read NGS for the capacity to detect the threshold of heteroplasmy, the aligned_sorted.bam file resulting from the alignment of samples ctrl1, del1 and del2, sequenced by either ONT Rapid Sequencing and ONT Ligation Kits, was exploited as input file for Mitoverse (2023) [https://mitoverse.i-med.ac.at], an analysis platform that provides several tools for the analysis of mtDNA sequencing reads. In particular, the mtDNA-Server was used for mtDNA variant detection; it can be executed locally or from the cloud web service of Mitoverse Platform (https://mitoverse.i-med.ac.at/). Once the aligned_sorted.bam file was loaded in the server, variants analysis was launched with default setting parameters. In the resulting file the following fields were present: annotated variants filtered keeping only the ones showing a good quality (PASS filter), variated base, heteroplasmy level and coverage depth.

#### Structural variants (SVs) calling

Alignment files generated by NGMLR were used as input for Sniffle to perform variant calling across both nuclear DNA and mitochondrial DNA. This produced an output.vcf file, which includes all SVs that were called by the default parameters except for the –s parameter defining the minimum number of reads to support the structural variant that was set to 2. For large deletions calling, Sniffle uses those reads mapping on mtDNA that contain a gap of several kb within their sequence, after being aligned to the reference mitochondrial genome. Only the reads spanning the breakpoints were used for calling large mtDNA deletions.

The VCF file was further processed to get selectively the SV calls (particularly, deletions) affecting the mitochondrial genome. To accomplish this, BCFtools has been used. BCFtools is a set of utilities that manipulate variant calls in the Variant Call Format (VCF) and its binary counterpart BCF (Danecek et al., 2021). Fields regarding chromosome name, alteration type, start and end positions of the SV breakpoint and number of reads supporting the variance have been extracted preferentially from the VCF file outputted from Sniffle. However, information exclusively concerning deletions affecting the mtDNA were collected, to further use them for Circos plots generation.

#### Circos plot generation

In order to allow the visualization of precise breakpoints positions of mtDNA large or multiple deletions as well as the potential presence of mtDNA complex rearrangements, Circos software package was used (Krzywinski et al., 2009). The tool was installed on a UNIX system from command line, following the installation instructions present at the developers’ website (Krzywinski et al., 2009). A main configuration file, written in HTML, is required to generate a Circos plot carrying the feature of interests. However, many additional files written with a specific format [as reported in the Circos tutorials (Krzywinski et al., 2009)] are needed to draw each plot component independently. For the purposes of this work, a circos plot for each sample analyzed was generated, containing a representation of the circular mtDNA molecule. Moreover, the generated plot was implemented to show the mitochondrial DNA encoded genes, the origins of replication of the mtDNA molecule, the coverage obtained from the sequencing run and the deletion breakpoints for patients affected by mtDNA structural alterations.

## Results

Library preparation was performed using the whole DNA extracted and purified from muscle biopsies of 16 different individuals. Two different ONT MinION sequencing kits were tested: the Rapid and the Ligation Sequencing kits**.**

### ONT ligation sequencing kit (SQK-LSK109)

#### Library preparations and sequencing data

This kit was tested for the sequencing of the whole DNA of a group of 12 samples (3 control samples, 9 samples carrying a single-large mtDNA deletion). Because of the circularity of the mitochondrial genome, a linearization step is required to allow the sequencing of the mtDNA molecules by the MinION platform. We tested 2 restriction enzymes, BamHI and PvuII: BamHI was reported in a previous paper for mtDNA sequencing using Nanopore chemistry (Bicci et al., 2021), while PvuII was the enzyme used in our laboratory for mtDNA linearization before SB analysis. Notably, these two enzymes cut the mtDNA molecule in highly distant positions: BamHI has a cleavage site at m.14262 and PvuII at m.2652.

All the 12 samples (ctrl1-3 and del1-9) were digested with BamHI enzyme and sequenced with the Ligation Sequencing Kit; among them, 8 samples were also linearized with PvuII restriction enzyme (ctrl1, ctrl2, del2, del3, del5, del7, del8, and del9) (Table 1). Once the enzymatic digestion and DNA purification by beads were performed, a sufficient amount of input DNA was obtained for all samples.

This kit allowed to obtain an average number of reads per sample of 167,815, an average read length of 4,398 bp, with the longest reads produced of 88,860 bp. Analyzing the reads mapped to the mtDNA, an average read length of 5,030 bp was obtained allowing to sequence the mtDNA with an average depth of coverage of 541x across the twelve samples. The minimum number of reads was obtained for sample del8 (47,888 reads) while the maximum value is reported for sample del2 (347,736 reads). Details and average numbers of reads per sample are reported in the Supplementary Table S1.

#### mtDNA coverage analysis and alterations detection

Raw reads mapped to the mtDNA by Minimap2 have been loaded into IGV to visualize alignment results and analyze the coverage profile of each sequenced sample.

**FIGURE 1:**
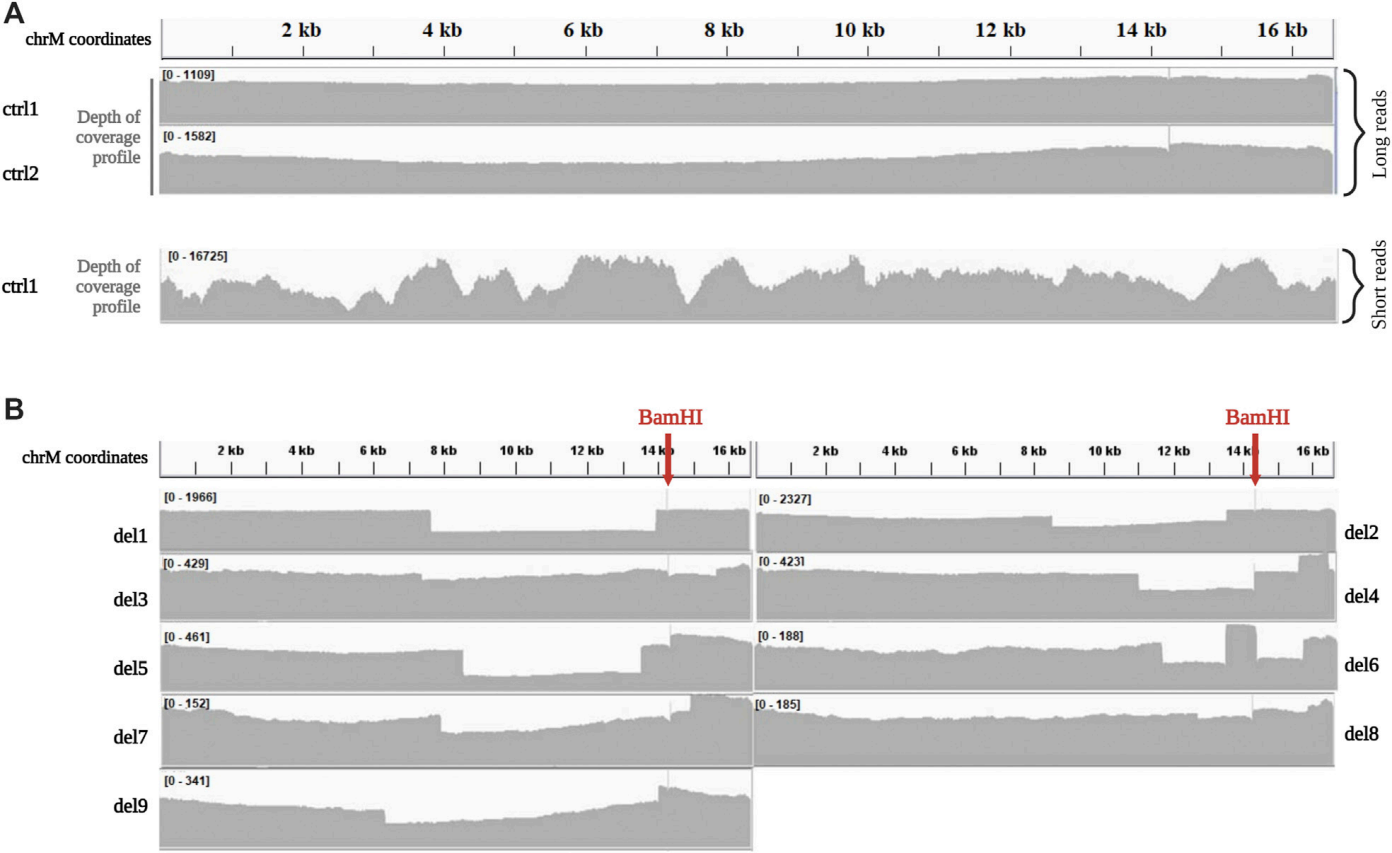
Depth of coverage of mtDNA long-read sequencing by the Ligation kit **(A)**. Depth of coverage and visualization by IGV of mtDNA for two control samples (ctrl1 and ctrl2) processed with the Ligation sequencing kit. Coverage profile for ctrl1 sequenced by short-reads NGS is reported for comparison. Data were obtained by processing single-amplicon PCR of the whole mtDNA according to Nextera XT protocol (Illumina), and sequencing mtDNA libraries on MiSeq platform (read length 2 × 150 bp, paired-end), as previously described (Legati et al., 2021). **(B)**. Depth of coverage and visualization by IGV of mtDNA for samples with single large deletions (del1, del2, del3, del4, del5, del6, del7 del8, and del9) processed with the Ligation sequencing kit.

In control samples (ctrl1, ctrl2), the reads produced by ONT Ligation Sequencing Kit after BamHI linearization showed a very homogeneous distribution of coverage along all the mitochondrial genome compared to the coverage of the whole mtDNA sequenced by short-read NGS (Figure 1A). A coverage spike was visible at the BamHI cleavage site (m.14262), since most of the reads start there.

Among samples with mtDNA macrodeletions, coverage profile of two (del1, del8) clearly highlighted the presence of a region affected by a decrease in the coverage with starting and ending positions easily identifiable (Figure 1B), and corresponding with deletion breakpoints detected by standard sequencing (Table 1). In the other 7 samples (del2, del3, del4, del5, del6, del7, and del9), coverage profile showed anomalies in the drop of coverage spanning the deletion (Figure 1B). Notably, these corresponded with samples were BamHI cleavage site falls into deletion coordinates. For example, samples del4 showed multiple drops in the coverage profiles, not compatible with the presence of a single large deletion; similarly, in sample del6 an unexpected peak in the depth of coverage was present within the region affected by the known single deletion.

**FIGURE 2:**
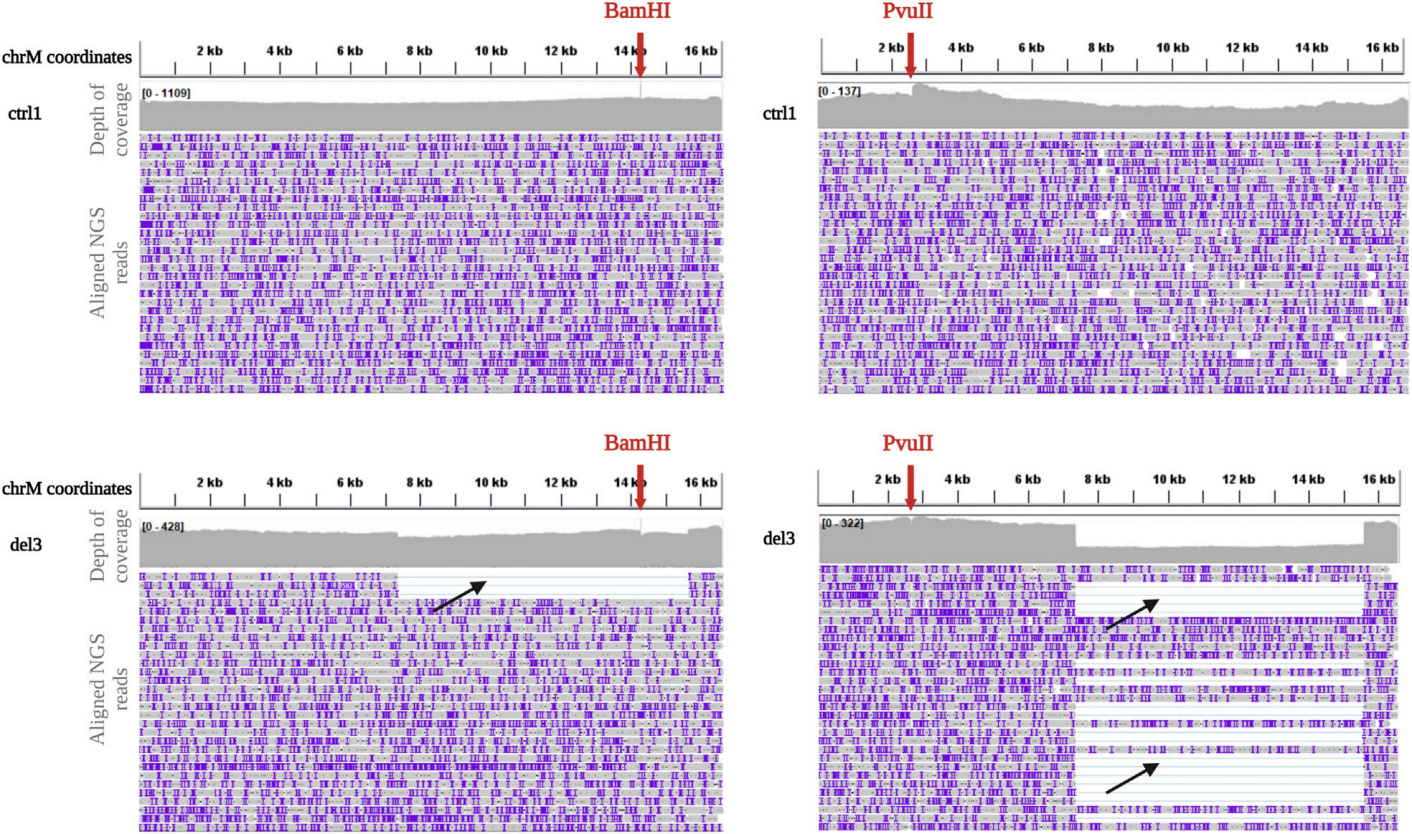
Depth of coverage and alignment visualization after BamHI and PvuII linearization. Depth of coverage and alignment visualization by IGV of mtDNA for a control (ctrl1, upper panels) and a sample with single deletion (del3, lower panels) after digestion with BamHI and PvuII (red arrows indicate the cleavage sites) and processing with the Ligation sequencing kit. Reads containing the deletion are clearly visible (black arrows).

Because of these problems with BamHI linearization, we tested another restriction enzyme, PvuII, currently used in our laboratory for SB analysis. PvuII cleavage site is at position m.2652, in a region which is spared in most of the known mtDNA single deletions. Eight DNA samples (ctrl1, ctrl2, del2, del3, del5, del7, del8, and del9) previously processed with BamHI were linearized by PvuII endonuclease, since its cleavage site falls outside their single large deletions. Digested DNAs were processed using the ONT Ligation Sequencing Kit and then sequenced on the MinION. The use of PvuII did not affect the uniformity of coverage in controls (Figure 2) and sequencing metrics (Supplementary Table S1). However, it allowed eliminating some biases in proper detection of the deletion. An example is reported in Figure 2, with a clear drop in the coverage spanning the deleted region for sample del2 processed by PvuII, while a less evident drop was present for the same sample processed by BamHI.

### ONT rapid sequencing kit (SQK-RAD004)

Given the possible problem associated with the selection of a specific restriction enzyme, we tested an alternative approach based on DNA randomly fragmented by transposase. The same approach was also evaluated for samples presenting multiple mtDNA deletions, which are usually spread along the mtDNA molecule, although frequently located within the major arc (i.e., the region between the origins of replication of heavy and light mtDNA strands) (Samuels et al., 2004).

#### Library preparations and sequencing data

The Rapid Kit was tested to sequence the whole DNA isolated from muscle biopsies of 6 individuals: one control sample previously sequenced by the Ligation Kit (ctrl1), 2 samples carrying mtDNA large deletion (del1 and del2) and 4 samples carrying mtDNA multiple large deletions (multidel1, multidel2, multidel3, and multidel4).

Analyzing the reads mapped to the mtDNA, an average read length of 3,574 bp and an average depth of coverage of 486x was obtained for samples sequenced on MinION Flow Cell R9.4.1 (ctrl, del1, and multidel1) while an average read length of 3,638 bp and an average depth of coverage of 134x was obtained by using the Flongle Flow Cell (multidel2, multidel3, and multidel4). Despite the mean global read length being homogeneous among samples, distribution of mtDNA read length is highly variable in samples with deletions, reflecting the coexistence of wild-type molecules and deleted molecules with different sizes. Details and average numbers of reads per sample are reported in the Supplementary Table S1, while the distributions of reads length for samples ctrl1, del1, and del2 processed by Rapid and Ligation kit are reported in Supplementary Figure S1.

#### mtDNA coverage analysis and alterations detection

**FIGURE 3:**
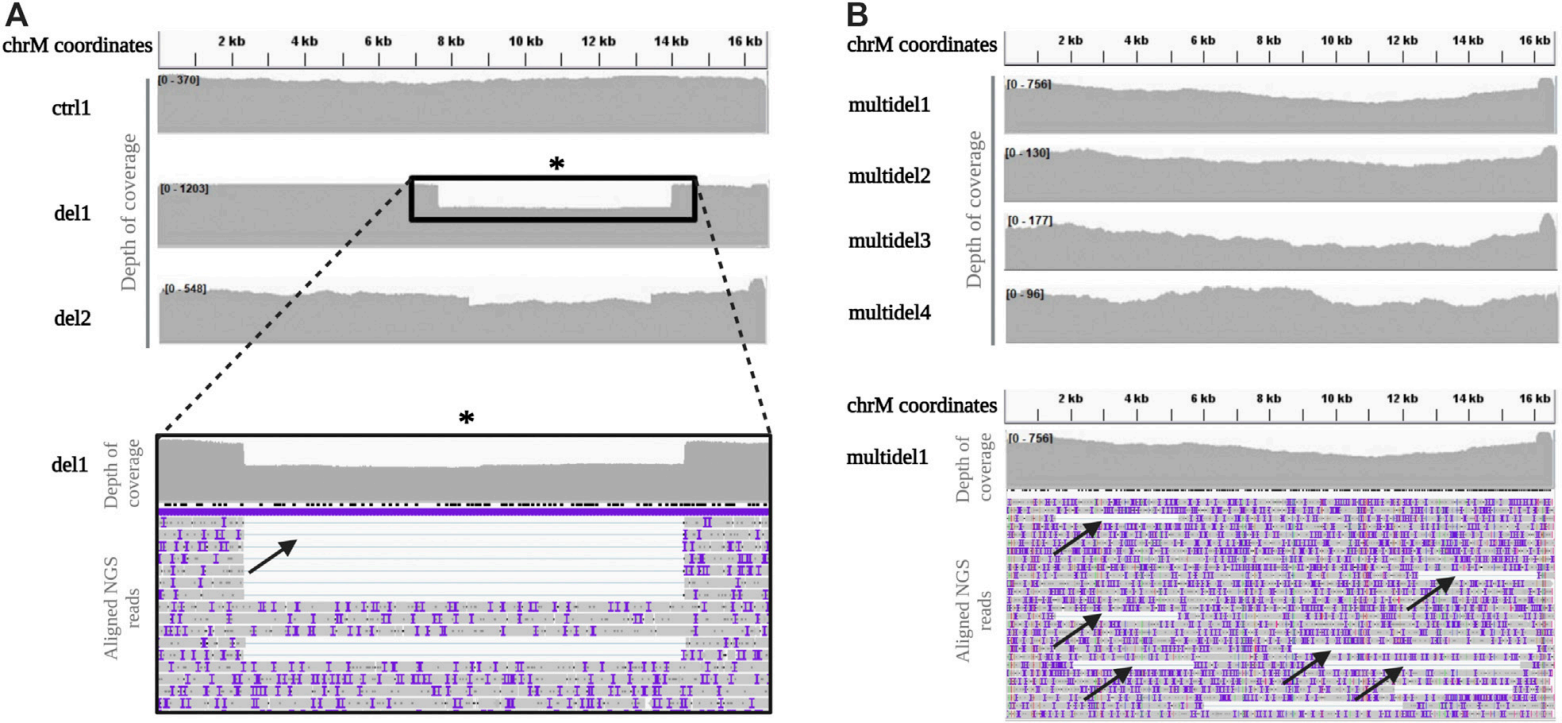
Depth of coverage and alignment visualization of mtDNA long-read sequencing by the Rapid kit **(A)**. Depth of coverage for a control (ctrl1) and two samples with single deletion (del1 and del2) sequenced using the Rapid Sequencing Kit. The box reports a detail of the mtDNA single large deletion in sample del1, easily identifiable by a drop in the coverage. **(B)**. Depth of coverage of mtDNA long-read sequencing for samples with multiple deletions (multidel1, multidel2, multidel3, and multidel4). Reads containing different deletions in the sample multidel1 are clearly visible (black arrows).

Even with the Rapid kit, homogeneous distribution along all the mtDNA was obtained in the control sample (Figure 3A). For samples with single deletion, coverage profile showed a visible drop in coverage depth spanning the deleted portion, clearly allowing identification of the deletion breakpoints (Figure 3A).

Concerning the mtDNA analysis of patients (multidel1, multidel2, multidel3, and multidel4) previously diagnosed by SB for carrying multiple deletions, coverage analysis did not evidence a clear drop in the coverage as for samples with single deletions. However, looking at the individual aligned reads, several different large deletions were visible (Figure 3B).

### Bioinformatics analysis of mtDNA rearrangements

#### Structural variants (SVs) calling

The detection of mtDNA structural alterations, such as large deletions and complex rearrangements, was performed by Sniffle SVs caller exploiting the mapping output obtained by NGMLR aligner.

In the 10 sequenced samples with single deletions, Sniffle analysis generated a vcf file for each sample, containing the starting and ending position of the single-large deletions and their sequencing depth (Table 1; Supplementary Table S2). Conversely, for the 4 samples presenting multiple mtDNA deletions, Sniffle generated vcf files with a list of deletions, their breakpoints and their sequencing depth.

**FIGURE 4:**
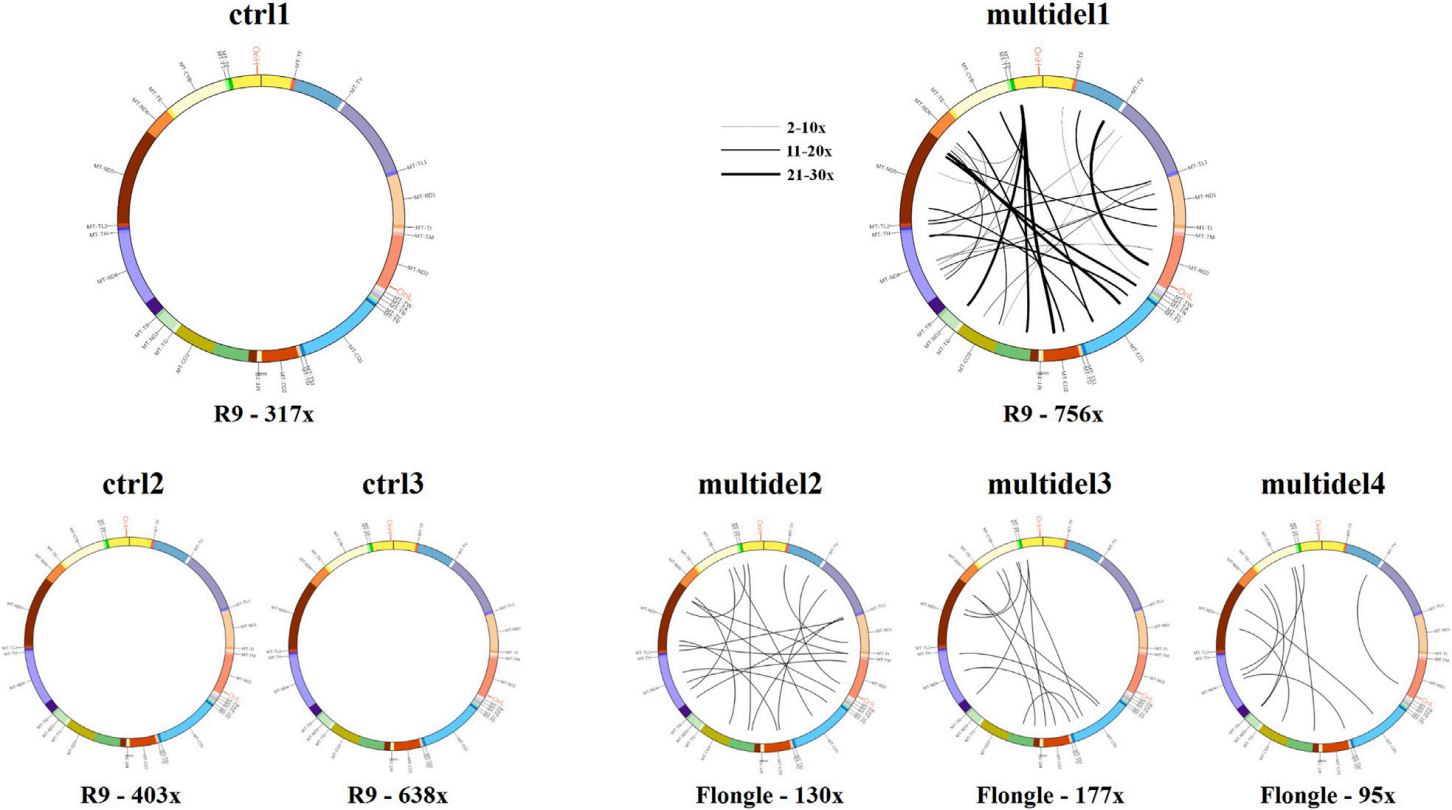
Circos plot visualization using Circos plots for control samples (ctrl1, ctrl2, and ctrl3) and samples carrying multiple mtDNA deletions (multidel1, multidel2, multidel3, and multidel4) sequenced using the Rapid kit. Black lines indicate the deletions and link the coordinates of deletion breakpoints. For sample multidel1 (sequenced on the Flow Cell R.9.4.1), lines thickness is proportional to the number of reads containing the deletion. Under each graph, flow cell type and average depth of coverage are indicated (R9 = Flow cell R9.4.1).

In order to simplify the interpretation of the output generated by Sniffle for the multiple deleted samples, a visual graph with the precise breakpoints positions of all the deletions was generated using the Circos software package. The presence of multiple deletions was evident when alignment data are plotted in circular graphs, with each line corresponding to individual identified deletions. Control samples showed no lines (i.e., no deletions), while multiple deleted samples were characterized by a dense pattern of lines linking deletions breakpoints. Moreover, the thickness of visualized lines was proportional to the number of reads with the same deletion (Figure 4).

#### Heteroplasmy quantification of mtDNA single deletions

Heteroplasmic percentages of mtDNA deletions were calculated for each sample carrying a single mtDNA deletion, by measuring the ratio between the average depth of coverage within the deletion and the average depth of coverage of the wild type mtDNA regions not affected by the deletion. The obtained values were compared to Southern blot data resulting to be similar for samples processed by both the Ligation (mean difference vs. SB: 13% ± 9%) and the Rapid kits (mean difference vs. SB: 14% ± 4%) (Table 1). Noteworthy, heteroplasmic percentages were calculated for sample del3 separately processed by BamHI and PvuII endonucleases. The mtDNA digested with BamHI showed a down estimated heteroplasmy (13%) of the deletion when compared to the same sample digested with PvuII (54%), or with the results obtained by standard techniques (Table 1).

To evaluate the possible influence of depth of coverage (and hence of the used flowcell type) on heteroplasmy calling, we generated for sample del1, del2, and del3 new bam files with reduced coverage (around 100x), using picard downsampling command. We obtained similar percentages for the single large deletion (max difference 10%) as measured in the original bam file (with coverage >400x) (Supplementary Figure S2).

The Circos visualization described above can be also applied to single deletions, irrespective of the library preparation kit used. Furthermore, this visualization confirmed the influence of the restriction enzyme used for mtDNA linearization for the Ligation Sequencing Kit (Supplementary Figure S3).

### Additional analysis of long-read NGS data

#### mtDNA sequencing

Although MinION sequencing accuracy is known to be not optimal, we exploited produced data to test sequencing performance for 3 control samples (ctrl1, ctrl2, and ctrl3); sample ctrl1 sequenced with the Rapid Sequencing Kit and samples ctrl1-3 sequenced by the Ligation Sequencing Kit.

We noticed that long-reads bare several errors due to the presence of small insertions and deletions (INDELS), which is an intrinsic limitation of Nanopore sequencing dynamics. In particular, ONT is prone to homopolymer run error (Dohm et al., 2020); anyway, the longest homopolymer in mtDNA is a poly-A of 8 bases (chrM:12,418-12,425). The accuracy of long-read NGS in the quantification of heteroplasmy was tested for single point mutations. The mean upper bound of the 95% asymmetric confidence interval of alternate reads for all the mtDNA positions in the control samples was 4.7% (±1.6%); the lower bound was 0% since most of the positions in all tested samples, including the median, presented only calls for the reference nucleotide (Supplementary Table S3). Short indels were excluded from the variant calling analysis and from error rate calculation. Although it is not the best approach for this purpose, these data indicate that ONT sequencing can be used for point variant calling if the requested heteroplasmy detection limit is not <5%. Accordingly, based on sequence accuracy, the error rate for ONT sequencing has been estimated to be around 4%–6% (Delahaye & Nicolas, 2021; Luo et al., 2022). Furthermore, Nanopore recently released new flow cells (R10.4.1) with increased accuracy which could further improve mtDNA sequencing (Luo et al., 2022).

#### Relative mtDNA quantification

Since the sequencing was performed on whole muscle DNA, we tested the possibility to exploit the data produced by the Nanopore Sequencing to check the relative amount of mtDNA respect to the total DNA. In particular, the mtDNA/nDNA number of reads ratio was calculated for all 13 samples sequenced by the Ligation Sequencing Kit and the 7 samples sequenced by the Rapid Sequencing Kit (Supplementary Table S4). This cohort had an intrinsic bias because calculating relative mtDNA amount for deleted samples could be affected by the presence of the heteroplasmic large mtDNA deletion.

Samples previously diagnosed by SB and short-read NGS for carrying a large mtDNA deletion, were also analysed by quantitative PCR (qPCR). We evaluated mtDNA quantity by normalizing the number of reads mapping on mtDNA with those mapping on nDNA in data obtained by MinION long reads with both Rapid and Ligation kits (Supplementary Table S4).

## Discussion

### Muscle tissue for mitochondrial disorders genetic diagnosis

MD patients affected by mtDNA large deletions can present heterogeneous clinical symptoms and disease onset; however, one of the most commonly affected tissues for this group of patients is muscle. Given the high energy consumption related to its activity, muscle tissue contains an elevated number of mitochondria and defects in their activity due to mtDNA alterations lead to a severe impairment of muscle functionality, causing myopathies. The biological mechanisms behind the generation and accumulation of mtDNA deletions among the different patients’ subgroups are not fully characterized.

**FIGURE 5:**
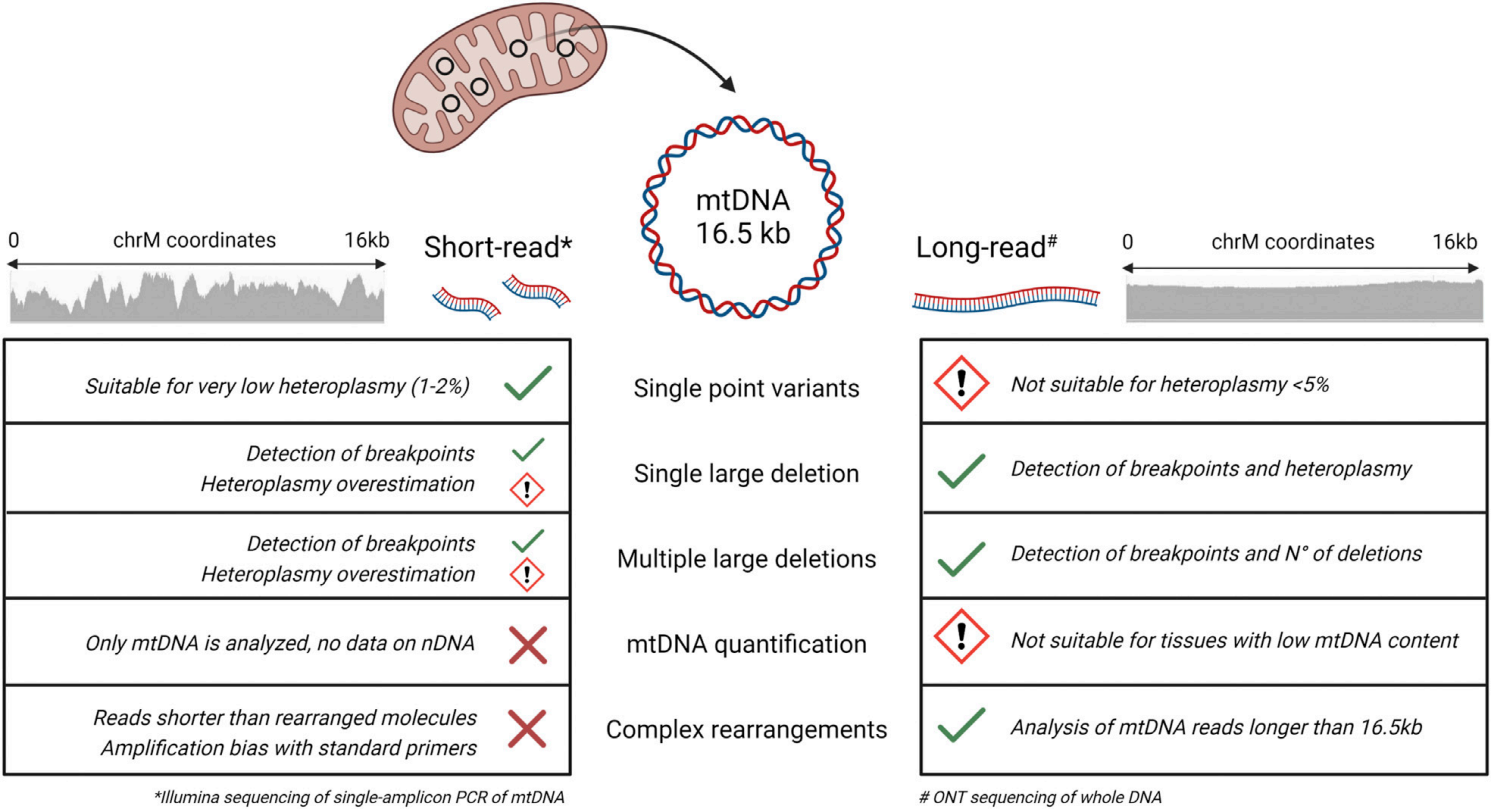
Short and long-read performance comparison for the sequencing of mtDNA. Summary of the performances of short-read (Illumina) and long-read (ONT) sequencing in the detection of different mtDNA alterations. For evaluation of short-reads, we used the single amplicon protocol described by (Legati et al., 2021), while for long reads we referred to the present study. Short-read NGS is suitable for identifying single point variants, even at very low heteroplasmic percentages, but the use of reads of 150–300 bp in length hampers the detection of large deletions and complex rearrangements. Long-read NGS shows multiple advantages in the analysis of mtDNA alterations, with some limitations regarding the minimum heteroplasmy detectable for single point variants and the quantification of mtDNA in tissues characterized by a lower content of mitochondria. (Image created with BioRender.com).

Traditionally, SB analysis and long-range PCR have been the methods of choice for the detection of mtDNA deletions. Recently, short-read NGS has become the standard approach for mtDNA sequencing and single point mutations detection. However, short reads have intrinsic limitations for the identification of mtDNA complex rearrangements and for the heteroplasmy quantification of large deletions (Legati et al., 2021) (Figure 5). For these reasons, there is a diagnostic need for appropriate methods to investigate mtDNA.

In this study, long-read NGS was tested on whole DNA extracted from patients’ muscle biopsies for detection of mtDNA structural alterations. We chose muscle tissue for two main reasons: the first one relies on the fact that in this tissue there is high abundance of mitochondria and of mtDNA; the second one is that muscle is the specimen where mtDNA deletions are most frequently present in patients with such genetic alterations. Given the high amount of mitochondria in muscle cells, total DNA extracted from these specimens is naturally enriched in mtDNA, hence sequencing the whole DNA leads to a large fraction of reads mapping to mtDNA. We obtained this expected finding with long-read NGS, using both the Rapid and Ligation kits, for all the samples analyzed. This allowed to subsequently have a good and homogeneous depth of coverage along the entire mtDNA molecule. The possibility of sequencing whole DNA samples represents a strong advantage since it does not require the availability of biological samples for mitochondrial isolation (typically fresh muscle tissue) and can be performed even on long-term stored DNA samples.

Considering the positive results, we plan to evaluate the possibility to analyze DNA samples isolated from other biological tissues, as alternative to muscle (e.g., human skin fibroblast, blood, urinary epithelial cells), exploit them for the screening of mtDNA large deletions and complex rearrangements. Validating these tissues for the analysis mtDNA could represent an alternative and noninvasive procedure able to reduce the discomfort for the patients who underwent a muscle biopsy and the associated costs in terms of money, time and personnel required. However, the lower content of mitochondria in these tissues is expected to lead to a low, possibly insufficient, number of reads mapping on mtDNA, respect to muscle samples, when analysing total DNA. To overcome this potential issue, a first easy option is to extend for longer time the sequencing run of DNA samples isolated from “low-mitochondrial content” samples. Indeed, the MinION device allows to monitor “in real time” the produced amount of reads mapping on mtDNA and to stop the run when the desired mtDNA coverage has been reached. For those samples for which extending sequencing run time would be not successful, we can envisage four possible approaches as potential solutions to increase the number of mtDNA reads. As first instance, enrichment of mtDNA molecule could be obtained by selectively degrading genomic DNA by an exonuclease treatment. An enzyme proposed to degrade linear DNA molecules is the exonuclease V that has different nuclease activities, comprising an ATP-dependent double-stranded and bi-directional exonuclease activity. Exonuclease V enzyme is able to deplete linear DNA fragments and thereby improve the proportion of circular mtDNA versus nuclear DNA [as described in the work by Jayprakash AD and others (Jayaprakash et al., 2015)]. A second strategy for enriching mtDNA is based on its selective amplification from total DNA. REPLI-g Mitochondrial DNA kit (Qiagen), which is based on the multiple displacement amplification technology (MDA), has been successfully used for this purpose (Vandiver et al., 2022). Recently, another option to selectively improve the number of mtDNA reads has become available. ONT has developed adaptive sampling (compatible only with the GridION MK1 device), which allows for simple enrichment by loading a FASTA file of a target region. By sequencing the first 400–500 bp of a DNA library, adaptive sampling software can identify reads containing target region or not. If not, the read is ejected from the pore by reversing the voltage (Mariya et al., 2022).

It is also possible to perform biological enrichment during the sample preparation, by the isolation of mitochondria and the subsequent extraction of DNA (Legati et al., 2021). However, this approach requires availability of a certain amount and quality of starting material, easily obtainable for cultured cells but not for other specimens (e.g., muscle biopsy, urinary epithelial cells). Moreover, the use of Cas9 technology has been lately described by two independent studies for the enrichment of mtDNA to obtain full-length human mitochondrial genomes as native single molecules (Vandiver et al., 2022; Keraite et al., 2022).

### Nanopore MinION sequencing performance

We tested two library preparation kits working with different proceedings. Their main features and differences are reported in Table 2, while further information and details on sequencing performances and reads parameters per sample are reported in Supplementary Tables S1, S2. The Rapid Kit allows to directly sequence whole DNA samples; DNA is randomly fragmented by transposase and tagged at the cleaved ends with adapter sequences. The Ligation Kit allows to optimize and increase the sequencing output. It requires enzymatic digestion with a single endonuclease able to cut mtDNA in a unique position, in order to linearize it. After this digestion, DNA ends are repaired and dA-tailed and then sequencing adapters are ligated onto the prepared ends.

**TABLE 2:** Comparison between the ONT ligation and ONT rapid sequencing kits.

	*Ligation Kit*	*Rapid Kit*
Average N° of reads per sample^a^	240,117	176,790
Average N° of reads mapped on chrM^a^	2,702	2,184
Average mtDNA read length^a^	6,220 bp	4,403 bp
Average depth of mtDNA coverage^a^	921x	545x
mtDNA linearization	Endonuclease (specific site)	Transposase (random)
mtDNA single large deletion	OK	OK
	Warning: endonuclease’s cutting site must fall outside the single large deletion	
mtDNA multiple deletions	OK	OK
	Warning: endonuclease’s cutting site can fall within multiple deleted regions introducing bias in their detection	
mtDNA complex rearrangements	NO	OK
	The linearization of mtDNA by endonuclease in a specific site can alter molecules containing complex alterations	

^a^ Average values were calculated from NGS, data of samples ctrl1, del1, del2 (R9.4.1 flowcell, no multiplexing).

By looking at the results of our work, we can state that the Nanopore sequencing allows to obtain a high (>100x) and homogeneous mtDNA depth of coverage with both the Rapid and Ligation Kit (Figures 1–5). With the classical MinION Flow Cell (R9.4.1), a single sample run lasts an average of 2.5–3 h, leaving several pores in the used flow cell still available for 1-2 further sequencing runs. Alternatively, it is possible to use the multi-sample sequencing by Native Barcoding DNA expansion pack: it allowed us to pool samples together to sequence them in a single MinION run. We loaded up to 8 samples at the same time with a run until exhaustion of the flow cell (about 20 h). Finally, we tested also the Flongle, low output flow cell: samples were loaded singularly running till exhaustion (about 7 h) and coverage output resulted significantly reduced (on average about 130x for mtDNA). Nevertheless, we showed that a depth of coverage around 100x is enough to obtain a reliable estimation of heteroplasmy for mtDNA macrodeletions.

In general, the Ligation Kit ensures a better output in terms of number of reads per sample and average read length. Both kits allowed us to detect start and ending positions of single large deletions and to quantify their heteroplasmic percentages. With the Rapid Kit it was possible also the identification of mtDNA multiple deletions (Figure 4).

Furthermore, we tested the possibility to evaluate mtDNA content quantifying the relative mtDNA vs. nDNA amount, potentially exploitable for detecting mtDNA depletion (Figure 5). Preliminary results are promising, but further experiments need to be performed, eventually analyzing also samples affected by mtDNA depletion.

Some additional considerations can be made regarding the sequencing performance obtained by the Ligation and Rapid Kits, respectively.

#### ONT ligation sequencing kit

During DNA library preparation steps, we started from a DNA concentration higher than the one suggested by the protocol, since during the many purification steps some DNA is inevitably lost. The increase of DNA concentration improved significantly the efficiency of the experiment, allowing us to load an amount of DNA equal or sometimes superior to that required by the protocol. All the samples sequenced by the Ligation Sequencing Kit produced a good output in terms of number of reads per sample, average read length and mtDNA depth of coverage (Figures 1, 3). An advantage of this kit is due to the fact that DNA ends are repaired and mtDNA is linearized at a specific site, leading to an increase of the sequencing yield and, consequently, of the mtDNA depth of coverage. However, linearization of mtDNA molecule occurring in this protocol can potentially give rise to biases due to the cleavage performed by the restriction enzyme selected. Consequently, the choice of the specific restriction enzyme to process the DNA relies on *a priori* knowledge of deletion breakpoints or the lack of it.

Since BamHI is widely used in published literature for linearizing mtDNA molecules, we decided to use it in our first experiments. However, BamHI cleavage for some samples resulted affecting the accuracy with which deletion breakpoints are delineated, as it is visible from coverage anomalies (Figure 1). Consequently, in cases in which deletion positions are unknown it is preferred to perform linearization by PvuII restriction enzyme, since its cutting site is more often found outside the deleted region. Furthermore, samples linearized by PvuII enzyme showed a higher number of reads mapped on mtDNA per sample compared to the samples linearized by BamHI enzyme.

We hypothesize that this phenomenon occurred because PvuII enzyme is less efficient in processing nDNA, compared to BamHI, creating a lower number of shorter fragments and thus favoring nDNA portions to be sequenced by the flow cell pores. However, mtDNA amount of reads as well as its average read length did not result to be affected.

If whole DNA is scarce for both quality and quantity, the Ligation kit offers the possibility to sequence PCR amplicons of the mtDNA molecule. However, performing NGS analysis of mtDNA amplicons generated by PCR can present some limitations, in particular heteroplasmy overestimation for large deletions and difficulties to identify complex rearrangements.

In conclusion, for the Ligation Kit the use of a specific endonuclease for linearizing mtDNA limits the identification of mtDNA complex rearrangements and requires choosing a restriction enzyme with a cleavage site that falls outside the mtDNA deletion.

#### ONT rapid sequencing kit

The random enzymatic fragmentation occurring on the mitochondrial genome allows the presence of deletions or putative structural rearrangements to be preserved so that precise information about mtDNA structure can be easily and reliably assessed through an accurate downstream bioinformatics analysis.

Due to the simple nature of the workflow and the fact that little sample manipulation is required, some very long reads have been achieved with this kit, despite the required transposase fragmentation. A quantity higher than the recommended input of DNA was used, allowing a better recovery of longer fragments. All the samples sequenced by the Rapid kit gave a good output in terms of number of reads per sample, average read length and mtDNA depth of coverage (Figures 1, 3). Moreover, since mtDNA is randomly fragmented, this kit gives the possibility to detect complex rearrangements and multiple deletions without bias affecting breakpoints and heteroplasmic percentages.

**FIGURE 6:**
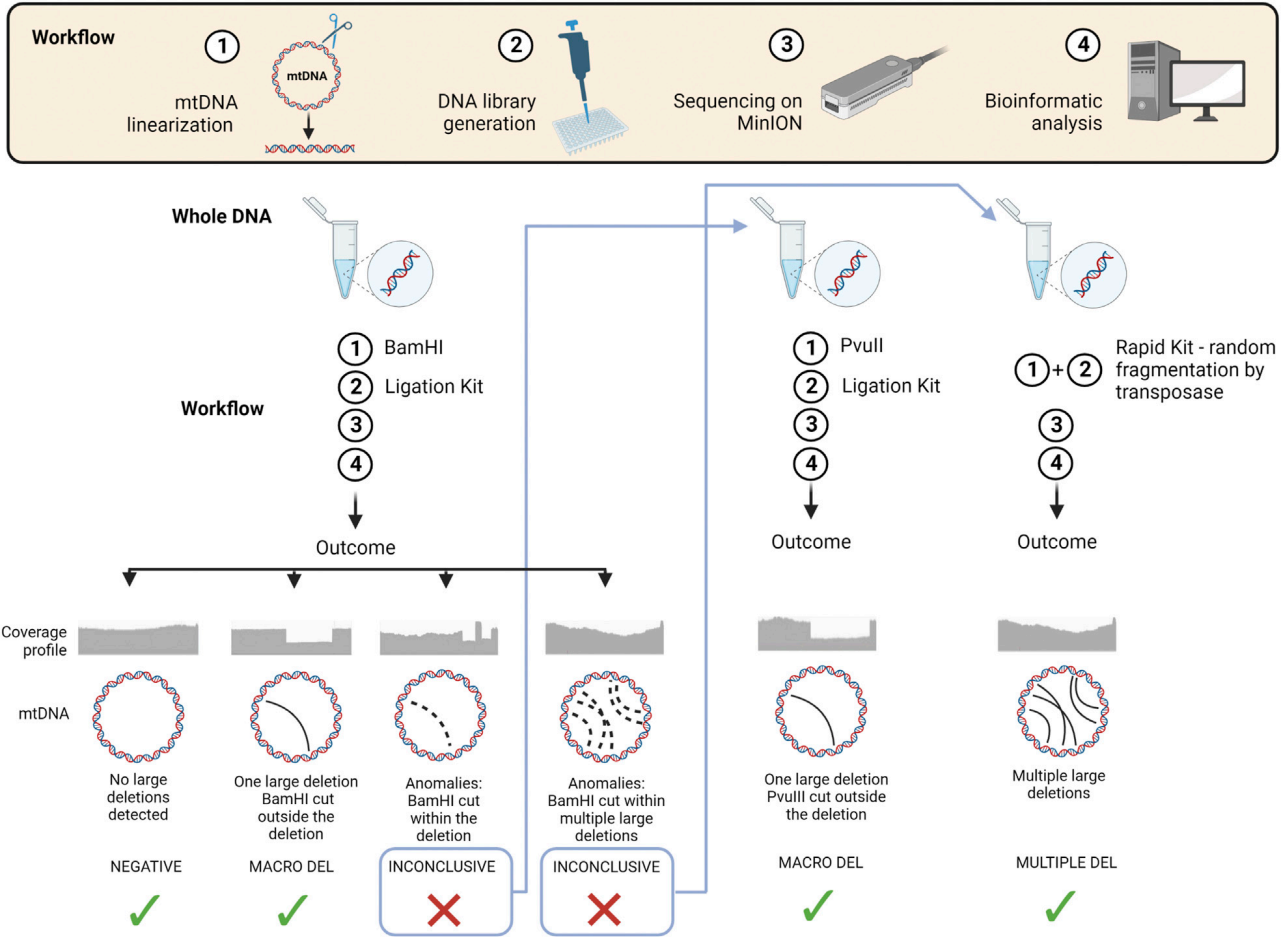
Schematic overview of the workflow and strategy proposed for long-read NGS in the analysis of mtDNA structural variants We summarize in a schematic view the 4 main steps of the workflow applied for sequencing mtDNA through ONT technology. We also propose an ideal strategy for exploiting its advantages based on our findings and experience. Starting from a new DNA sample, we suggest, as first approach, to linearize the mtDNA using BamHI endonuclease and then process the DNA using the Ligation Kit. Four potential outcomes can be obtained from data analysis: (i) a negative sample where no large deletions are detected, (ii) a positive sample with a single large deletion, (iii) a sample with anomalies due to the localization of BamHI cutting site within the single large deletion, (iv) a sample with coverage anomalies due to multiple large deletions. In the first two cases the analysis can be considered concluded, while in the third case we recommend to re-sequence the DNA sample after linearizing it with PvuII endonuclease, and in the fourth case to re-sequence the sample using the Rapid Kit, which randomly fragments mtDNA using a transposase. (Image created with BioRender.com).

Based on our findings and experience with the two tested kits, we propose a workflow for mtDNA structural variant analysis (Figure 6). We suggest the ligation kit as the first approach, using BamHI to linearize the mtDNA. If no large deletions or a single large deletion is detected, the analysis can be considered concluded. Some samples may display anomalies due to the localization of BamHI cutting site within the single large deletion, or to the presence of multiple large deletions. In the first case, we recommend re-sequencing the DNA sample after linearization with PvuII. In the latter case, it is better to re-sequence the sample using the Rapid Kit, which randomly fragments mtDNA by a transposase.

### Long-read NGS for diagnosis in mtDNA-related diseases

The establishment of a diagnosis in patients with suspected MD is often a challenge. Traditionally, the diagnosis of MD has been based on demonstrating mitochondrial dysfunction in a relevant tissue biopsy (e.g., skeletal muscle), with the specific pattern of biochemical abnormalites being used to direct targeted molecular genetic testing of mtDNA, specific nuclear genes, or both (Chinnery, 2021).

Nowadays, genetic testing is the most reliable way to diagnose and categorize a mitochondrial disorder. Muscle biopsy is the current gold standard for the measurement of heteroplasmy to predict the incidence of specific clinical features. However, in recent years several laboratories have investigated mtDNA alterations using a range of noninvasive tissues, including hair follicles, fibroblasts, buccal mucosa, and urinary epithelium. For instance, back to 2004 it was reported how in patients with the m.3243A>G mutation urinary epithelium cells carry the most consistent mutation load if compared to the heteroplasmy detected in muscle biopsies (McDonnell et al., 2004).

Alterations of mtDNA molecule have been studied for a long time by traditional techniques (e.g., Sanger sequencing, Southern blot, and qPCR). However, these technologies are limited in speed, throughput, and sensitivity. Sanger method had been the gold standard for DNA sequencing, particularly for small DNA regions and single genes (Legati et al., 2021), until some years ago, when it was substituted by NGS. However, in some less advanced laboratories its use could be still part of the diagnostic routine. Sanger sequencing has a low detection sensitivity for mtDNA heteroplasmy level (Carroll et al., 2014). Precise detection of large deletions and assessment of mtDNA depletion are not suitable for this method. SB analysis can identify mtDNA species affected by single or multiple deletions, without giving information regarding heteroplasmy level, as well as position of deletion breakpoints (Legati et al., 2021). qPCR is employed in the measurement of mtDNA copy number, providing also information about mtDNA level of heteroplasmy for single macrodeletions (Rooney et al., 2015). Measurements by qPCR for detecting mtDNA depletion require the use of a reference nuclear gene and are often displayed as a ratio of mitochondrial to nuclear DNA. However, qPCR is particularly susceptible to different PCR efficiencies between target and control samples, leading to skewing of this ratio.

Currently, NGS is used in clinical practice to study mtDNA alterations (Figure 5). Short-read based NGS of single mtDNA amplicon is suitable for generating full accurate mtDNA sequence, assessing heteroplasmy for single point mutations with high accuracy, and detecting break positions of single large deletions (Legati et al., 2021). Additional approaches suggested enrichment of mitochondria before DNA isolation (Legati et al., 2021) or the use of exonuclease V to eliminate or reduce nuclear DNA before sequencing. However, short-reads (200 bp) are limited for the identification of structural mtDNA alterations, due to the lack of coverage uniformity and the limited size of the reads themselves.

A further element complicating the use of short-read NGS for mtDNA analysis is the presence of nuclear DNA of mitochondrial origin (NUMTs), i.e., mtDNA fragments inserted in nuclear genomic sequences during evolutionary process in eukariotes (Richy and Leister, 2004). The human DNA contains >700 NUMTs and several NUMTs may correspond to the same mtDNA region (Dayama et al., 2014) making their influence on mtDNA analysis a non-trivial issue. Most human NUMTs are longer than reads produced by short-read NGS, hence sequence alignments cannot distinguish with certainty mtDNA from NUMTs, generating possible false negative or false positive calling, and error in heteroplasmy quantifications (Singh et al., 2021). However, a recent study (Wei et al., 2022) conducted on whole-genome sequences from 66,083 people and focused on elucidating the complex NUMT landscape, evidenced how there is a weak correlation between germline NUMT mtDNA breakpoints and the location of known deletion breakpoints in mtDNA. This data suggests that the risk of calling false positive mtDNA large deletion due to the alignment of genomic NUMTs on mitochondrial genome is very low.

Long-read NGS, including the technology based on the MinION sequencer, can overcome the limitations of short-read approaches for the analysis of mtDNA optimizing an NGS strategy able to detect large mtDNA deletions and complex rearrangements in mitochondrial disease patients (Figure 5). ONT sequencing is still characterized by limitations in accuracy for single variant calling, in particular for homopolymeric stretches. Other long-read sequencers, such as the PacBio HIFI, seem to be superior to ONT for this aspect, given the lower error rate in reads generated with this technology. However, we focused this study on detection of mtDNA rearrangements rather than mtDNA sequencing; furthermore, we tested a cheap sequencer, the MinION device, which can be easily used in most laboratories. Additionally, the really short time of DNA library preparation and of the sequencing itself are a big advantage compared to traditional techniques present in clinical practice for the analysis of the mitochondrial genome even when compared to short-read NGS. However, all the potential of long-read NGS, which allows generating sequences with the size of the entire mtDNA molecule, has not yet been fully defined.

In conclusion, third-generation sequencing technologies based on long-reads are a valuable approach to study structural alterations in mtDNA, which represent a causative genetic defect in a subset of MD and usually require long and cumbersome analyses for their identification. As also indicated by the promising results obtained in our work, long-read NGS can be potentially exploited to easily investigate by a single method and innovative data analysis pipelines all the mtDNA genetic defects associated with MD.

## Data Availability

The original contributions presented in the study are available here https://doi.org/10.5281/zenodo.7273735, after clarifying the intended use of data.
