# Supplementary material for: Nanopore long-read next-generation sequencing for detection of mitochondrial DNA large-scale deletions
Source: Front Genet. 2023 Jun 29;14:1089956. doi: 10.3389/fgene.2023.1089956 (PMC10344361; doi:10.3389/fgene.2023.1089956)
Supplement: Supplementary file 1 [file DataSheet1.pdf]

Supplementary Table 1: metrics of long-read sequencing.

Statistics obtained using samtools stats command on bam files generated by minimap2.

Rapid kit					
Sample	N° of reads per sample	Global read length	mtDNA read length	mtDNA depth of coverage	Notes
ctrl1	89742	3395	4349	317	Flow Cell R9.4.1
del1	177313	3984	6365	935	Flow Cell R9.4.1
del2	263317	3664	2496	384	Flow Cell R9.4.1
multidel1	245610	4107	3876	756	Flow Cell R9.4.1
multidel2	32643	3953	5213	130	Flongle Flow cell
multidel3	52645	2300	2461	177	Flongle Flow cell
multidel4	77275	2638	3242	95	Flongle Flow cell

Ligation kit	N° of reads per sample		Global read length		mtDNA read length		mtDNA depth of coverage		Notes
Sample	BamHI	PvuII	BamHI	PvuII	BamHI	PvuII	BamHI	PvuII	
ctrl1	263277	70910	4285	2168	4981	3058	639	137	BamHI + PvuI
ctrl2	260629	141985	2850	2680	2636	3500	1582	403	BamHI + PvuII
ctrl3	231800	\	2466	\	2422	\	638	\	BamHI
del1	109337	\	6541	\	8519	\	957	\	BamHI
del2	347736	126470	4379	2941	5160	6308	1167	427	BamHI + PvuII
del3	93676	211055	4581	4506	4100	3907	429	322	BamHI + PvuII
del4	100248	\	5803	\	6109	\	423	\	BamHI
del5	91820	272438	3968	3968	4906	5502	461	653	BamHI + PvuII
del6	79424	\	2916	\	2821	\	100	\	BamHI
del7	61384	292553	4255	5759	4940	5759	152	708	BamHI + PvuII
del8	47888	249439	4860	9270	5737	9270	185	844	BamHI + PvuII
del9	73700	146250	3454	5508	4177	5508	340	445	BamHI + PvuII

Supplementary Table 2: mtDNA reads containing the deletions.

Statistics obtained from bam file generated by ngmlr and deletions calling using sniffle.

Rapid kit

Sample code	N° of reads mapping on chrM	N° of reads with deletions	% of reads with deletions
ctrl1	1624	0	0,0%
del1	4052	326	8,0%
del2	3308	65	2,0%
multidel1	3298	264	8,0%
multidel2	473	18	3,8%
multidel3	500	47	9,4%
multidel4	919	11	1,2%

Ligation kit

	N° of reads mapping on chrM		N° of reads with deletions		% of reads with deletions	
Sample code	BamHI	PvuII	BamHI	PvuII	BamHI	PvuII
ctrl1	3017	567	0	11	0,0%	1,9%
ctrl2	6559	1418	0	6	0,0%	0,4%
ctrl3	2543	/	2	/	0,1%	/
del1	3820	/	555	/	14,5%	/
del2	5736	1280	350	71	6,1%	5,5%
del3	1476	1312	24	139	1,6%	10,6%
del4	1097	/	93	/	8,5%	/
del5	1328	2068	156	251	11,7%	12,1%
del6	857	/	43	/	5,0%	/
del7	500	2170	30	337	6,0%	15,5%
del8	528	2498	12	352	2,3%	14,1%
del9	969	1349	48	89	5,0%	6,6%

Supplementary Table 3: error rate in Single Nucleotide Variants calling.

	Base call error rate (confidence interval 95%)		Percentage of mtDNA bases called with an error rate lower than	
	Lower bound	Upper bound	5%	0%
Ctrl1 BamHI	0%	6,2%	96,0%	77,5%
Ctrl1 PvuII	0%	1,9%	99,3%	87,5%
Ctrl1 Rapid	0%	4,5%	97,7%	84,5%
Ctrl2 BamHI	0%	6,2%	95,8%	77,6%
Ctrl2 PvuII	0%	4,4%	97,7%	85,7%
Ctrl3 BamHI	0%	4,9%	97,5%	82,5%

Three different control samples (ctrl), prepared with the Rapid kit (ctrl1) and the Ligation kit (ctrl1, ctrl2, ctrl3) using different restriction enzymes (BamHI and PvuII) were evaluated. Error rate refers to calling of base different from the reference nucleotide. Only "PASS" variants (i.e., variants passing quality control metrics) were considered for error rate evaluations; short indels were excluded from this analysis.

Supplementary Table 4: ratio of mtDNA/nDNA number of reads.

Statistics obtained using samtools idxstats command on bam files generated by minimap2.

Rapid kit

Sample code	gDNA	mtDNA
ctrl1	111759	1591
del1	206796	4037
del2	317181	3157
multidel1	293860	2936
multidel2	35894	433
multidel3	62324	826
multidel4	88686	474

mtDNA/nDNA ratio
0,014
0,020
0,010
0,010
0,012
0,013
0,005

Ligation kit

	BamHI		PvuII	
Sample code	nDNA	mtDNA	nDNA	mtDNA
ctrl1	321026	3073	89189	589
ctrl2	311767	6778	178901	1525
ctrl3	286599	2585	\	\
del1	230324	3924	\	\
del2	430711	5510	147495	1205
del3	109765	1620	241765	1203
del4	117423	1118	\	\
del5	109671	1158	319697	1819
del6	72245	475	345182	1859
del7	94790	850	\	\
del8	56684	530	289845	2134
del9	88083	927	171265	1268

mtDNA/nDNA ratio	
BamHI	PvuII
0,010	0,007
0,022	0,009
0,009	\
0,017	\
0,013	0,008
0,015	0,005
0,010	\
0,011	0,006
0,007	0,005
0,009	\
0,009	0,007
0,011	0,007

Supplementary Figure 1: distribution of reads length mapping on mtDNA.

A)

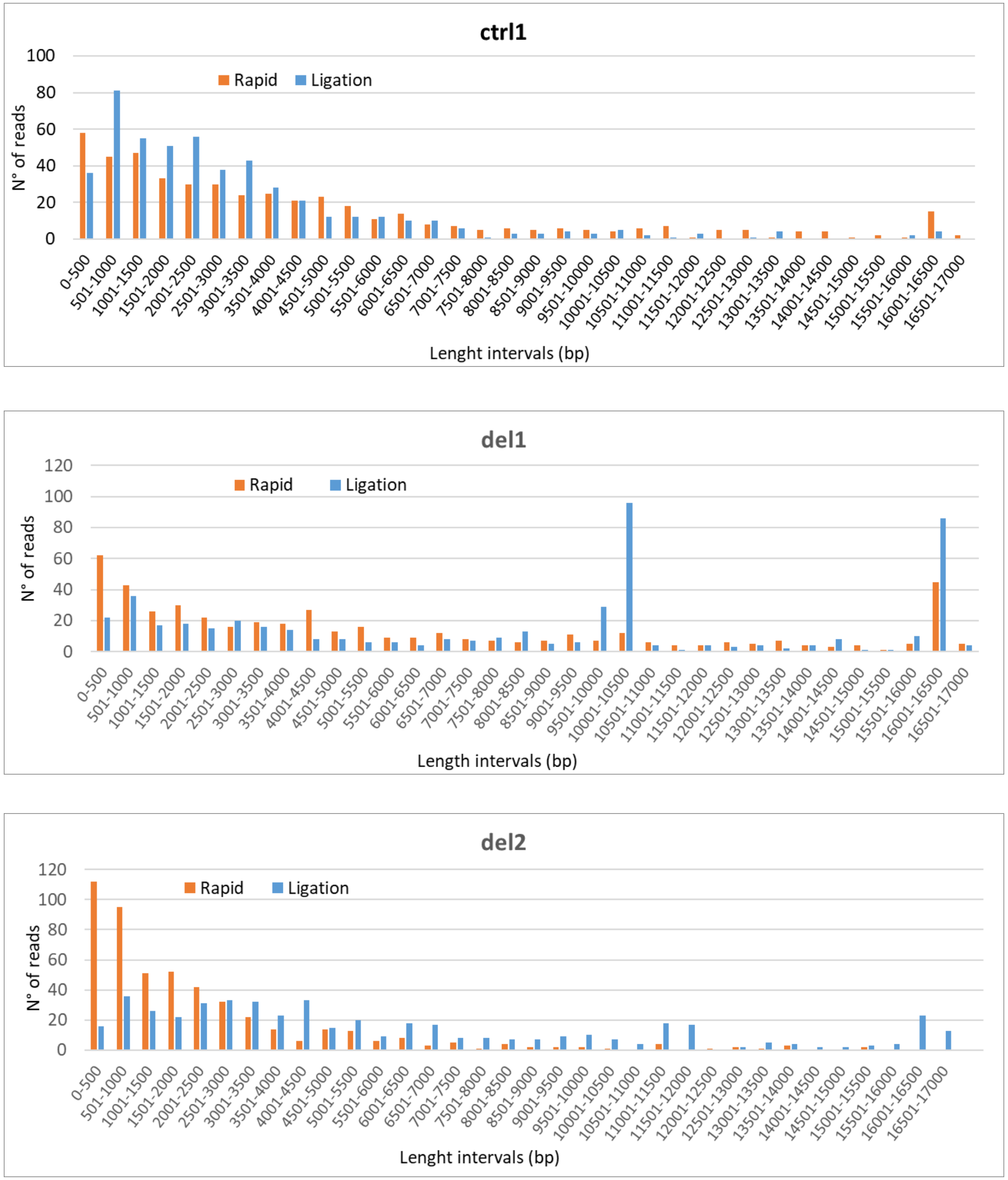

B)

Read N50 (bp)*

	Rapid	Ligation
**ctrl1**	2974	2282
**del1**	4135	9906
**del2**	1415	4364

Supplementary Figure 1. Analysis of lengths distribution of reads mapping on mtDNA for ctrl1, del1, del2 samples (values obtained for N° 500 reads per sample mapping on mtDNA).
(A) Reads lenght distribution comparison between the same sample sequenced with rapid and ligation kit. **(B)** Read N50 values comparison for the three samples sequenced with both rapid and ligation kit.

Supplementary Figure 2: heteroplasmy detection of single large deletions after *in silico* downsampling.

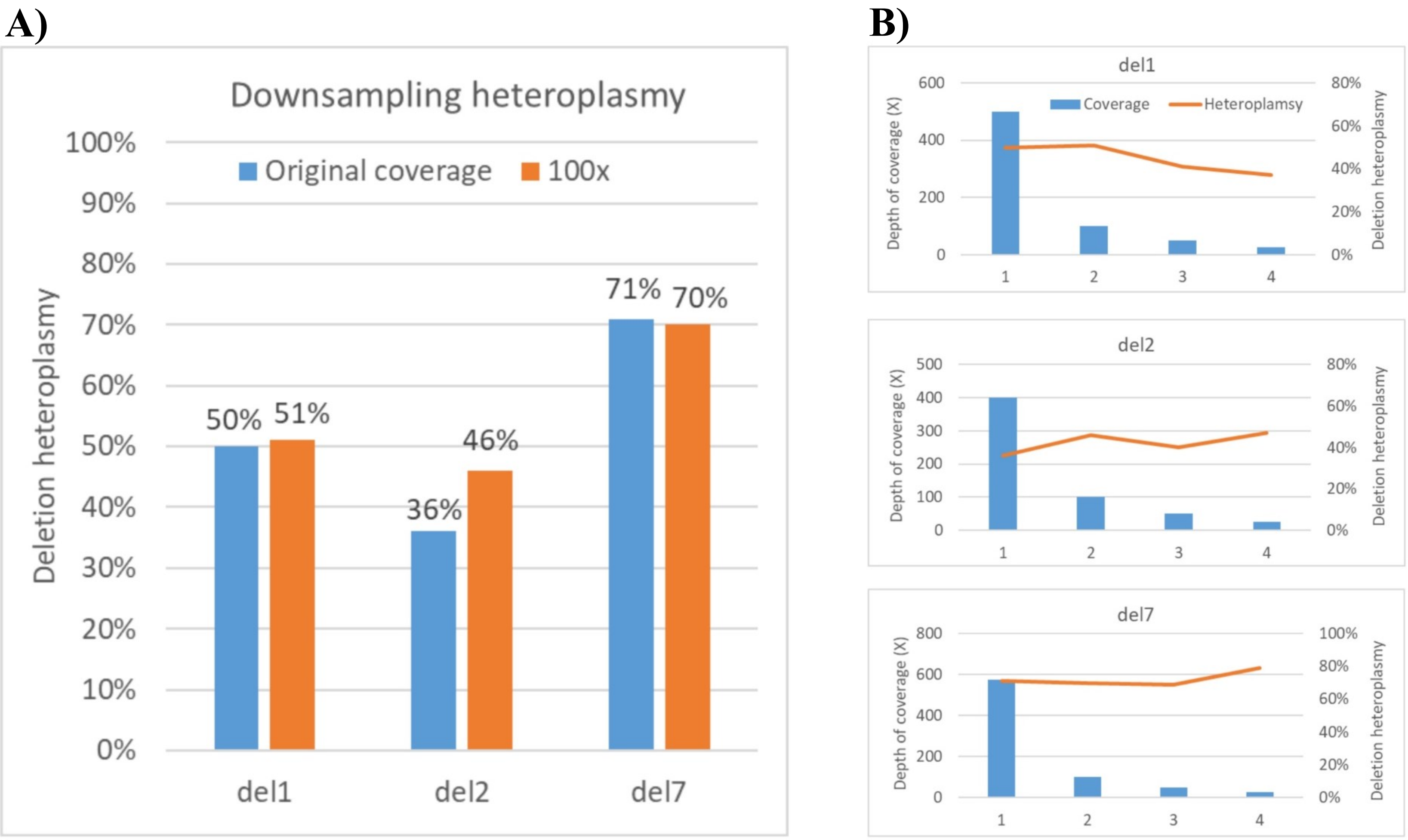

Supplementary Figure 2. (A) Deletion heteroplasmy percentages for the three samples (del1, del2, del7) affected by a single large deletion, calculated through the ratio between the average depth of coverage within the deletion and the average depth of coverage of the wild type mtDNA regions not affected by the deletion. For each sample, we compared the percentages obtained from the original alignment file and from a new alignment file (average mtDNA coverage 100x) generated thorugh an in silico downsampling (picard). (B) The same in silico approach was furtherly performed for downsampling the three samples also at 50x and 25x of average mtDNA coverage. Bars correspond to depth of coverage in the original file (1) or after downsampling at 100x (2), 50x (3) and 25x (4). The orange line indicates the deletion heteroplasmy in the four different conditions.

Supplementary Figure 3: Circos Plot for Ligation Sequencing Kit.

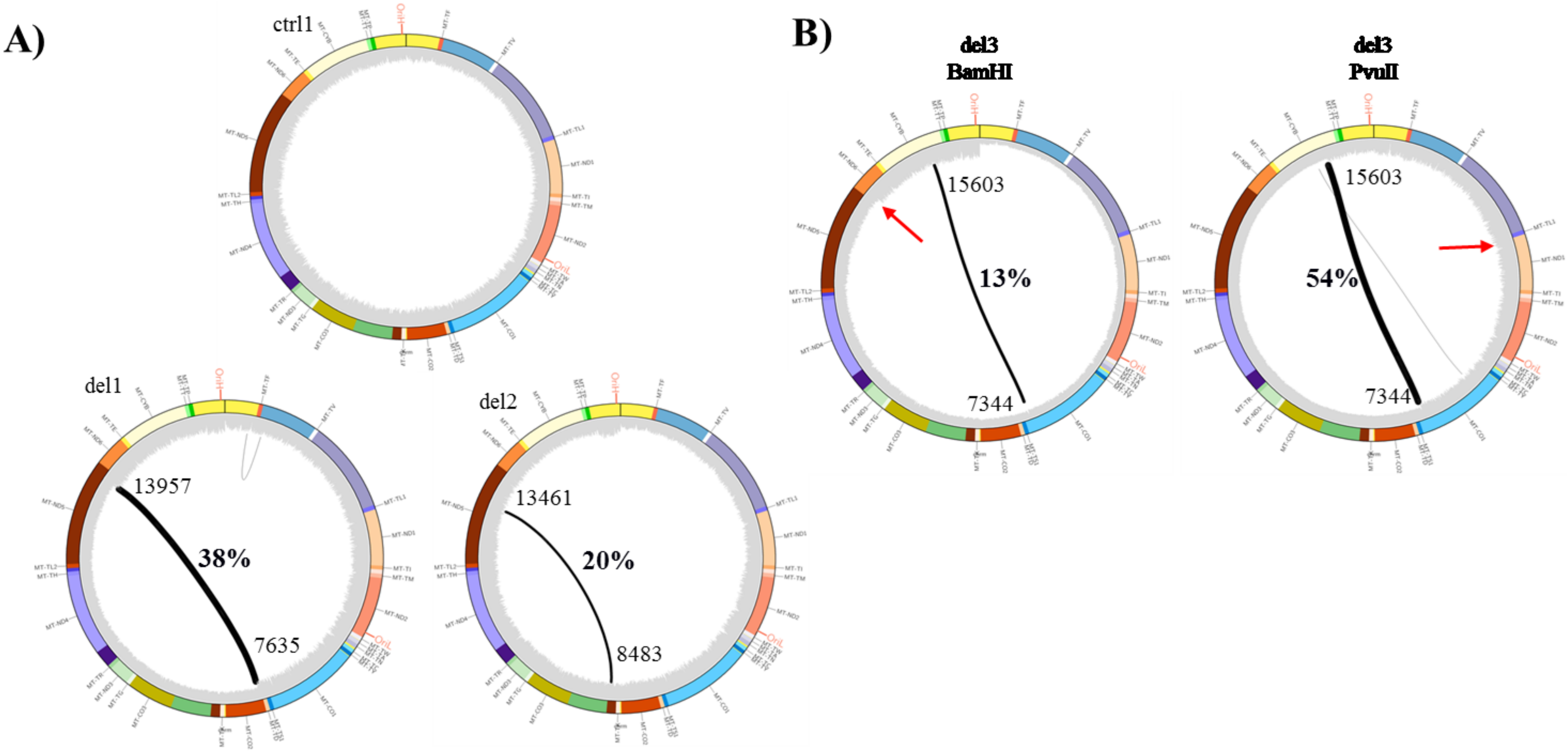

Supplementary Figure 3. (A) Summary of the circos plots obtained for 3 samples (ctrl1, del1, del2) sequenced using both Nanopore Rapid and Ligation Sequencing kits. Black lines indicate the deletions and link the coordinates of deletions breakpoints, lines thickness is proportional to the heteroplasmic percentage of the deletions. Coverage Profile is represented in gray.
(B) Sample carrying a single large mtDNA deletion (del3), sequenced using the Nanopore Ligation Sequencing Kit (mtDNA linearized using two different endonucleases, BamHI and PvuII); arrows indicate the cleavage sites of the endonucleases BamHI and PvuII. Black lines indicate the deletions and link the coordinates of deletions breakpoints, lines thickness is proportional to the heteroplasmic percentage of the deletions. Coverage Profile is represented in gray.
